# A VNTR Regulates miR-137 Expression Through Novel Alternative Splicing and Contributes to Risk for Schizophrenia

**DOI:** 10.1038/s41598-019-48141-0

**Published:** 2019-08-13

**Authors:** Ashley Pacheco, Ralph Berger, Robert Freedman, Amanda J. Law

**Affiliations:** 1 University of Colorado, School of Medicine, Department of Psychiatry, Aurora, CO 80045 USA; 2 University of Colorado, School of Medicine, Department of Medicine, Aurora, CO 80045 USA; 3 University of Colorado, School of Medicine, Department of Cell and Developmental Biology, Aurora, CO 80045 USA

**Keywords:** Genetics of the nervous system, Genetics, Risk factors

## Abstract

The *MIR137HG* gene encoding microRNA-137 (miR-137) is genome-wide associated with schizophrenia (SZ), however, the underlying molecular mechanisms remain unknown. Through cloning and sequencing of individual transcripts from fetal and adult human brain tissues we describe novel pri-miR-137 splice variants which exclude the mature miR-137 sequence termed ‘del-miR-137’ that would function to down-regulate miR-137 expression. Sequencing results demonstrate a significant positive association between del-miR-137 transcripts and the length of a proximal variable number tandem repeat (VNTR) element. Additionally, a significantly higher proportion of sequenced transcripts from fetal brain were del-miR-137 transcripts indicating neurodevelopmental splicing regulation. *In*-*silico* results predict an independent regulatory function for del-miR-137 transcripts through competitive endogenous RNA function. A case-control haplotype analysis (n = 998) in SZ implicates short VNTR length in risk, with longer lengths imparting a protective effect. Rare high risk haplotypes were also observed indicating multiple risk variants within the region. A second haplotype analysis was performed to evaluate recombination effects excluding the VNTR and results indicate that recombination of the region was found to independently contribute to risk. Evaluation of the evolutionary conservation of the VNTR reveals a human lineage specific expansion. These findings shed further light on the risk architecture of the miR-137 region and provide a novel regulatory mechanism through VNTR length and alternative *MIR137HG* transcripts which contribute to risk for SZ.

## Introduction

Schizophrenia (SZ) is a neurodevelopmental disorder with complex genetic etiology. To date, 145 genetic loci have been associated through genome wide association studies (GWAS) with adult onset schizophrenia (AOS)^1–4^. The microRNA-137 (miR-137) gene (*MIR137HG*) is a top association in these studies with four regional SNPs (rs1625579, rs1198588, rs2660304, rs1702294) reaching genome wide significance. The morphological and functional complexity in the brain depends on a highly coordinated gene expression program that includes small non-coding RNAs such as microRNAs (miRs) which are critical for neurodevelopment^5^. MicroRNAs predominantly function to down-regulate the expression of target genes by binding to mRNA and either targeting transcripts for degradation or preventing translation. Convergent evidence from genetic, phenotypic, and functional studies support the association of miR-137 with SZ, with miR-137 gain-of-function implicated in risk. Notably, there is a significant overlap between miR-137 target genes and genes associated with SZ^2,3,6,7^. *MIR137HG* risk genotypes are associated with phenotypic variability in AOS, such as age of onset, neuroanatomical structure, and function and negative symptoms of cognitive subtypes^8–11^. Aberrant neurodevelopment in-utero is thought to contribute to SZ, long before the onset of clinical symptoms. This is congruent with miR-137’s association to risk and biological role in neurogenesis and early neural development, with expression of miR-137 being tightly balanced during these critical windows^7,12^. miR-137 acts as a gene network hub during synaptic development, functioning upstream in many critical neurodevelopmental pathways that are independently associated with SZ such as Nrg/ErbB, PI3K-Akt-mTOR, and MAPK/ERK signaling^13–15^. Additionally, miR-137 regulates adult neurogenesis, synaptic plasticity, and glutamatergic signaling, all processes thought to contribute to the pathobiology of SZ^7,16–18^. Despite these numerous associations, specific molecular mechanisms underlying miR-137’s genetic association to SZ are unclear.

A single miRNA can regulate expression of thousands of target genes, and indeed miR-137 has over 1,300 predicted targets^19^. Therefore, even subtle changes in miRNA expression and function may have far reaching consequences. miR-137 is processed canonically through several stages and expression of miRs is largely dependent on processing efficiency^20^. Initially the primary miR (pri-miR) is transcribed and capped and tailed. The pre-miR sequence forms a stem-loop which is cleaved by the microprocessor comprised of DROSHA and its cofactor DGCR8. The pre-miR transcript is then exported out of the nucleus and cleaved a second time to become the final mature miR-137 strand. Determining how common genetic variants affect transcription and processing of miR-137 is a critical step in understanding molecular mechanisms contributing to the etiology of SZ. One variant found within *MIR137HG* which is proposed to alter miR-137 function, is a variable number tandem repeat (VNTR) element located within the pri-miR-137 transcript six bases upstream of the pre-miR-137 sequence. The VNTR is a repetitive sequence of 15 bases with high GC content. Three repeats is the shortest known length and the common allele. *In*-*vitro* studies show that mature miR-137 expression is affected by the length of the VNTR where greater lengths are associated with lower expression^21^. Variations in VNTR length have also been shown to affect Stroop test performance in healthy controls^22^. To date, the relationship between VNTR length and transcript variation of pri-miR-137 has not been examined.

The present study investigates whether the *MIR137HG* VNTR is associated with pri-miR-137 transcriptional variation during human brain development and contributes to the risk architecture of SZ through case-control haplotype analysis. The main objectives were (i) to characterize the transcription of MIR137HG at both early and late developmental time points through cloning and sequencing of human brain RNA, (ii) to evaluate VNTR length associations within transcript sequences, and (iii) to conduct haplotype analyses to evaluate whether the VNTR contributes to haplotype association with diagnosis. Our clinical cohort consisted of 307 healthy controls, 602 AOS patients, and an additional 89 childhood onset schizophrenia (COS) patients. COS is a rare form of SZ defined by the onset of psychotic symptoms before the age of 13^23^. Previous work has determined COS to be a more genetically severe form of SZ, associated with a higher rate of copy number variants in genes associated with pathways of neuronal development and regulation^23^. Evaluating genetic associations in multiple populations including COS is important for understanding the pathobiology of AOS and etiological differences between AOS and COS phenotypes. We predicted that GWAS SNP associations with AOS may also contribute to risk for COS, and therefore replicated SNP analysis in our COS cohort. Using a combination of molecular and bioinformatic approaches to evaluate *MIR137HG* transcription, we describe haplotype associations with SZ diagnosis, novel splice variants associated with VNTR length, and present new regulatory mechanisms for miR-137 which may have contributed to the evolution of increased human cognitive abilities as well as risk for complex neurodevelopmental disease.

## Results

### Transcript characterization

**Figure 1:**
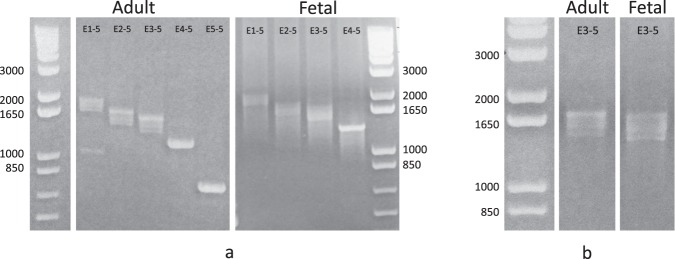
Pri-miR-137 PCR Exhibits Multiple Banding Pattern. (**a**) PCR results are shown for pri-miR-137 custom primer pairs in adult and fetal total brain cDNA. Exon primer pairs are labeled along the top of each gel lane (i.e. E1-5 is primer pair exon 1 to exon 5). Ladders and corresponding base sizes are shown on either side. Results for both adult and fetal total brain tissues depict correct product lengths for all primer pairs (E1-5:2034, E2-5:1797, E3-5:1694, E4-5:1307, E5-5:838). A consistent multiple banding patterns can be seen for all reactions containing exon 3, indicating alternative splicing within exon 3. (**b**) A magnified view of PCR results for primer pair E3-5 demonstrates potential differential transcript expression between time points with brighter bands of expected size relative to shorter bands in the adult tissue and equal brightness between band sizes in the fetal tissue. (**a**) Images were cropped from separate gels. (**b**) Images cropped from the same gel. Uncropped gel images are presented in Supplemental Fig. S1.

Transcription of pri-miR-137 was characterized using custom PCR primers in cDNA generated from pooled samples of human fetal and adult total brain RNA. Gel electrophoresis of PCR products exhibited a consistent multiple banding pattern for all reactions containing exon 3 (exon 1-5, exon 2-5, exon 3-5) with smaller product bands observed in addition to bands of expected size (Fig. 1a, Supplementary Fig. S1). For these reactions, amplicon base sizes ranged from approximately 350 bases less than to expected sizes (E1-5:2034, E2-5:1797, E3-5:1694). Single bands were seen for exon 4-5 and exon 5-5 amplifications (base size: E4-5:1307, E5-5:838). The observed multiple banding pattern indicates alternative splicing of pri-miR-137 transcripts within exon 3. When comparing the same primer reactions (and equal concentrations of cDNA) between fetal and adult tissues, we noted that PCR bands of expected size were brighter relative to shorter bands within the adult brain, whereas all bands exhibited apparent equal luminosity within the fetal brain (Fig. 1b, Supplementary Fig. S1). This qualitative observation indicates potential differential transcript expression in adult vs. fetal brain.

**Figure 2:**
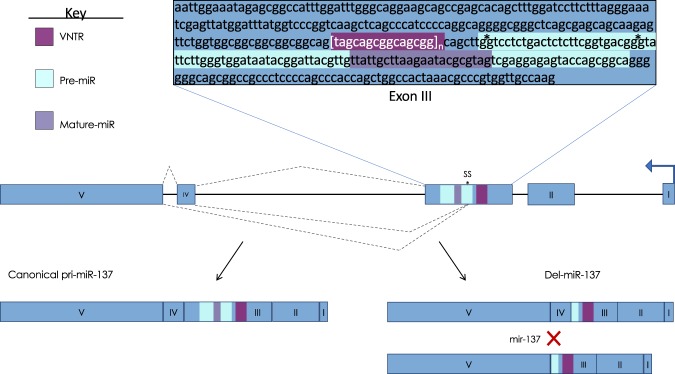
Novel Splicing of Pri-miR-137. The upper box contains the sequence of pri-miR-137 exon 3 with the splice sites indicated by asterisks. Canonical and alternative 5′ splicing of exon 3 is indicated by dashed lines above and below the region, respectively. Resulting canonical pri-miR-137 and del-miR-137 transcript isoforms are depicted along the bottom. Del-miR-137 transcripts do not contain the majority of pre-miR-137 sequence or the mature miR-137 sequence, which would down-regulate mature miR-137 levels. Expression of del-miR-137 transcript isoforms provides a novel regulatory mechanism for mature miR-137.

Next, cloning and sequencing of the PCR primer products allowed us to quantify and analyze relative proportions of pri-miR-137 transcript isoforms in the fetal and adult brain. Fifty-six total cDNA clones and corresponding full-length transcripts were sequenced from fetal brain and 78 total cDNA clones were sequenced from adult brain (Table 1). Two novel 5′ splice donor sites within pri-miR-137 exon 3 were identified, termed the primary (SS′) and secondary (SS″) splice sites based on frequency. The SS′ was found in 91% (30/33) of fetal spliced transcripts and 95% (n = 18/19) of adult spliced transcripts; the remainder of spliced transcripts were spliced at SS″. The remainder of cloned transcripts were confirmed as canonical pri-miR-137. Therefore, novel spliced transcripts accounted for 70% of the total in fetal brain and only 26% in adult brain. The location of both splice sites is within the pre-miR-137 sequence, upstream of the mature miR-137 sequence (Fig. 2). Both splice sites precede splice site consensus sequence GT nucleotides, consistent with alternative splicing as opposed to microRNA processing. Importantly, the resulting spliced transcripts exclude the mature miR-137 sequence and are thus termed ‘deleted-miR-137’ (del-miR-137). Processing efficiency and expression of miR-137 would be decreased through expression of del-miR-137 transcripts. This alternative splicing pathway for del-miR-137 reveals a previously unknown mechanism for the potential down-regulation of mature miR-137. Del-miR-137 sequence data was deposited to GenBank and assigned the following accession numbers: MK631885, MK631886 and MK631887.

**Table 1:** Cloning and Sequencing Transcript Data.

Variant	Adult Brain		Fetal Brain		FET p
	(n = 73)	Freq.(%)	(n = 47)	Freq.(%)	
SS’	18	24.7	30	63.8	
SS”	1	1.4	3	6.4	
Total SS	19	26.0	33	70.2	2.15e-06
VNTR Length	(n = 73)		(n = 56)		
3	66	90.4	30	53.6	
4	1	1.4	20	35.7	
5	0	0.0	2	3.6	
6	0	0.0	2	3.6	
7	5	6.8	2	3.6	
9	1	1.4	0	0.0	
Total >3	7	9.6	26	46.4	5.91e-06

SS’ - primary splice site, SS” - secondary splice site.

### Statistical transcript analyses

#### VNTR length frequency between age groups

The VNTR allele frequency between fetal and adult groups was significantly different with a higher proportion of transcripts containing minor (>3R) VNTR lengths in the fetal group (FET p = 5.919e-06) (Table 1). Differences in length of the VNTR between groups may reflect random population sampling differences between age groups.

#### Frequency of spliced transcripts between age groups

**Figure 3:**
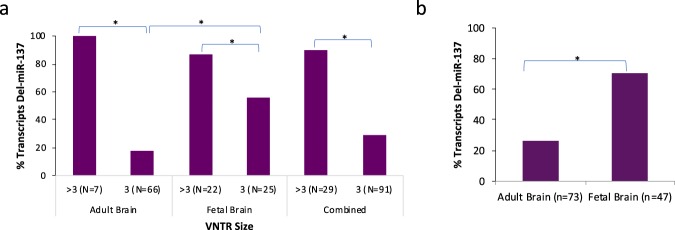
VNTR Length vs. Del-miR-137 Transcript Frequency. (**a**) Frequency of spliced del-miR-137 transcripts are depicted for fetal, adult, and combined allele length groups. There is a significant positive association between VNTR length and del-miR-137 frequency in all three (Adult, Fetal, Combined) groups (Adult: Fishers p = 3.09e-5, Fetal: Fishers p = 0.03, Combined: Fishers p = 7.10e-9). This correlation supports a role for VNTR length in alternative pri-miR-137 splicing, and ultimately regulation of miR-137 expression. Interestingly, there is also a significant difference in del-miR-137 frequency of the 3R transcripts between developmental groups with a higher frequency of 3R transcripts spliced in the fetal group (3R Fishers p = 6.8e-4). This indicates a potential epigenetic component underlying alternative splicing and that it is neurodevelopmentally regulated. (**b**) The frequency of del-miR-137 transcripts was significantly higher in the Fetal group (Fishers p = 2.15e-06) compared to the Adult group. We suspect that this reflects both an epigenetic developmental difference as well as differences in VNTR lengths between groups.

Next, the frequency of spliced transcripts was evaluated. All transcripts that spanned the splice sites (fetal 47/56, adult 73/78) were included in the frequency analysis. The proportion of spliced transcripts was significantly higher (FET p = 2.145e-06) in the fetal brain group(70%) compared to the adult group(26%) (Table 1, Fig. 3b). We propose that this finding is in part due to the VNTR length association with splicing frequency (Table 1) in addition to developmental regulation, as discussed subsequently.

#### VNTR length correlation with del-miR-137 splicing

Direct sequencing of cloned transcripts from fetal and adult total brain cDNA allowed us to evaluate VNTR length proportions between developmental groups and VNTR length associations with alternative splicing within and at an individual transcript level. All transcripts spanning both the VNTR and the splice sites were included in analyses of VNTR length versus alternative splicing (adult n = 73, fetal n = 47, combined n = 120). Importantly, we found significant positive associations between VNTR length and proportions of spliced transcripts, in both the adult and fetal brain. When transcripts were grouped by VNTR allele (3R, >3R), for both age categories as well as combined total transcripts, a significantly higher proportion of transcripts containing VNTR lengths >3R were also del-miR-137 transcripts (FET p = 0.029 fetal, p = 3.093e-05 adult, p = 7.098e-09 combined) (Fig. 3a). These results demonstrate novel correlations between VNTR length and alternative splicing of the pri-miR-137.

#### Splicing frequency comparisons between 3R groups across age

All transcripts containing the VNTR were included in VNTR allele frequency analyses. While it is notable that the VNTR allele frequency between fetal and adult groups was significantly different, between group analysis confirmed that the major VNTR allele (3R) in the fetal brain was associated with higher levels of del-miR-137 transcripts compared to the 3R in the adult group (FET p = 6.790e-04; Fig. 3a). This finding reflects possible developmental differences in epigenetic regulation of the region, independent of VNTR length, since VNTR genotype is consistent between groups. This observation is consistent with the previous finding of significantly higher del-miR-137 transcripts in fetal brain compared to adult (Fig. 3b). All findings remain significant after controlling for multiple comparisons using the Benjamini-Hochberg procedure.

Taken together, these results implicate VNTR length in conjunction with developmentally regulated epigenetic factors as part of a novel miR-137 regulatory mechanism mediated through pri-miR-137 alternative splicing.

### CpG island prediction

The VNTR sequence has high GC content and in order to evaluate contributions of VNTR length to regional GC content we analyzed putative CpG islands (CGIs) using EMBOSS. Results show two putative CGIs within the *MIR137HG* genome region one smaller 247 base CGI spanning HG19 chr1:98456956 – 98457203 and a larger 647 base CGI spanning HG19 chr1:98457215 – 98457862 (Supplementary Fig. S2). These regions overlap a large portion of pri-miR-137 exon 3, including the VNTR, pre- and mature miR-137 sequences and span into intron 3. Because the VNTR is within the CGI, increased VNTR lengths increase length of the CGI. These results indicate that the VNTR may impart regulatory effects on pri-miR-137 splicing through altered regional GC content.

### Competitive endogenous RNA *in silico* analyses

The del-miR-137 transcript constitutes a novel long non-coding RNA (lncRNA) that may have independent regulatory function. LncRNAs can regulate miRNA target gene networks through a competitive endogenous RNA (ceRNA) mechanism^24^. CeRNAs function by containing microRNA response elements (MREs) that competitively bind and sequester miRNAs^24^. To assess potential ceRNA functionality of the del-miR-137 transcripts, we first evaluated predicted MREs within the del-miR-137 sequence and generated a list of associated miRs using miRDB.org^25^. We found predicted MREs for 56 miRs (Supplementary Table S1). Next, we evaluated potential target genes regulated by the top scoring miRs with target scores >60 (n = 31). A list of 1,591 previously validated target genes was generated from the predicted binding microRNAs^26^. Pathway analysis and ranking of target genes determined ‘Axon guidance’ as the top associated pathway with 10 of the predicted del-miR-137 binding miRs known to target 45/266 total genes in the term (Supplementary Tables S1 and S2). Axon guidance pathway genes have been previously associated with risk for SZ^27^. These results indicate potential ceRNA function for del-miR-137 transcripts which may serve to developmentally up-regulate axon guidance associated pathways through sequestration of associated miRs.

### Analysis of GWAS SNPs in childhood onset schizophrenia

We found no significant association between SNPs rs1625579 (*χ*^2^ = 2.259, df = 2, p = 0.294), rs2660304 (*χ*^2^ = 1.195, p = 0.580) and COS diagnosis. SNP rs1198588 genotype was significantly associated with COS diagnosis (*χ*^2^ = 6.171, p = 0.048). The COS group had a higher frequency of minor (A/A) genotypes compared to controls (OR = 3.23, 95% CI 1.15–8.96). Interestingly, this is the opposite finding from GWAS of the adult SZ population where the risk allele is the major allele (T). Our individual variant analyses show no association between genotype at SNPs rs1625579 and rs2660304 and COS diagnosis.

### Case-control variant genotyping

The VNTR was genotyped in all patients and controls to evaluate association with diagnosis. VNTR lengths ranged from 3 to 14 repeats (Supplementary Table S3). Previous findings as well as our current findings for VNTR length frequencies, particularly for longer repeats, vary between studies^21,22,28,29^. We found through extensive troubleshooting that extended VNTR lengths (>9) require a high GC content PCR system in order to consistently amplify. We suspect that disparate allele frequencies between studies are due to different amplification protocols. Results of the VNTR case-control analysis failed to reach significance (p = 0.06), however the direction of effect indicates that the 3 repeat allele is found more frequently in patients (OR: 1.22), and we predict a significant effect may be seen with a larger sample size. Additionally, analysis of VNTR length alone is confounded by haplotype background, and potential additional risk variants within the region, we therefore next included the VNTR within a haplotype analysis.

### Haplotype analyses

**Figure 4:**
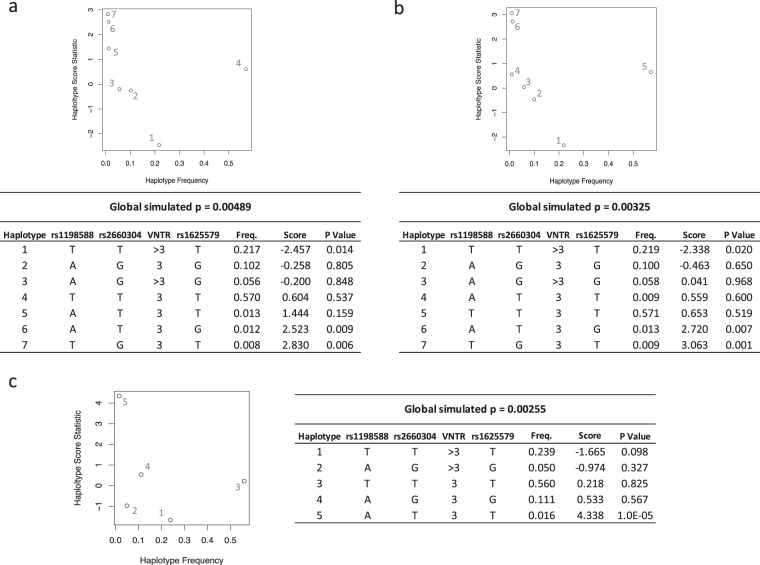
Global simulated p-value, haplotype frequencies, haplotype scores and simulated p-values are shown for Combined (**a**), AOS (**b**) and COS (**c**) Diagnoses. Global haplotype statistics are significant for all groups confirming an overall pattern of association with this haplotype region. The pattern of risk scores shows a potential protective effect for the TT > 3T haplotype, and a potential risk associations with the AT3G and TG3T haplotypes for the combined (**a**) and AOS (**b**) groups. The AT3T haplotype had a significant risk score in the COS group (**c**) indicating a potential contribution to more severe phenotypes. These results demonstrate a pattern of epistatic effects with both protective and risk haplotypes contributing to risk architecture.

To fully assess the VNTR contribution to risk while controlling for genetic background in the region, haplotype analyses were performed using SNP and VNTR genotyping data. This analysis also allowed us to evaluate risk for more rare haplotypes. Results are shown in Fig. 4. The common haplotype (TT3T) frequency for each group is: 54.7% in controls, 57.3% in the combined patient sample, 57.5% in AOS and 56.5% in COS patients. The global haplotype analysis was significant for all diagnosis groups (Combined global simulated p = 4.9e-03, AOS global simulated p = 3.3e-03, COS global simulated p = 2.6e-03) indicating overall association of the haplotype block with SZ. Tables and plots of haplotype risk scores for each diagnostic group are shown in Fig. 4. Haplotype risk scores indicate a significant protective effect for the (TT > 3T) group in the combined (p = 0.014) and AOS (p = 0.020) groups. In the COS group, the directionality of the protective effect of the (TT > 3T) was consistent but failed to reach significance (p = 0.098) likely due to the smaller sample size and lack of power in this group. Furthermore, significant haplotype associations to risk were also seen for three rare haplotypes. The combined and AOS groups showed significant association to the rare AT3G (Combined p = 0.009; AOS p = 0.007) and TG3T (Combined p = 0.006; AOS p = 0.001) haplotypes. The COS groups also had a significant score for the rare haplotype (AT3T p = 1.0e-5). Together, these results confirm overall association within the *MIR137HG* region to SZ and suggest combinatorial effects of the VNTR and the GWAS SNPs leading to both protective and rare high risk haplotype associations in patients with AOS and COS.

Results of the individual haplotype analysis allowed us to assess magnitude and direction of effect for significant haplotypes (Table 2). We used the protective haplotype (TT > 3T) as the reference to evaluate risk effects for the major haplotype. Results are consistent with the global haplotype score results. For the combined diagnosis group, individual haplotype analysis found significant effects for the common haplotype TT3T(OR: 1.32, p = 0.035), as well as for the rare haplotype AT3G(OR: 10.49, p = 0.022), and the rare haplotype group(OR: 2.36, p = 0.024). When the minimum frequency is lowered to 0.8% to allow for evaluation of the TG3T haplotype, complete separation is seen in the model with the TG3T haplotype only seen in cases and not in controls, and the rare haplotype group is no longer significant (p = 0.507). This complete separation confirms the global haplotype result that the TG3T haplotype is a rare high risk haplotype and indicates that the TG3T haplotype is driving the risk association with the rare haplotype group. An increased sample size is further needed to better assess the TG3T haplotype within the individual GLM model. Similar results were found for the AOS diagnosis group with significant risk associations for haplotypes TT3T(OR: 1.34, p = 0.034), AT3G(OR: 11.81, p = 0.016), and the rare haplotype group(OR: 2.22, p = 0.013). With the minimum haplotype frequency lowered to 0.9%, again we see complete separation with the TG3T haplotype being only found in patients, and the rare haplotype group becoming non-significant (p = 0.219). The risk association of the common TT3T haplotype relative to the TT > 3T haplotype implicates the major VNTR 3R allele as a driver of risk for the TT3T haplotype. Genotyping allowed us to conduct LD analysis of the VNTR and GWAS SNPs within our control cohort. Results indicate low linkage between the VNTR and GWAS SNPs (VNTR LD versus: rs1198588 D′ = 0.03, r = 0.02; rs2660304 D′ = 0.04, r = 0.03; rs1625579 D′ = 0.02, r = 0.01) suggesting that VNTR instability is not haplotype dependent, and that GWAS SNPs would not be tagging the VNTR. This is consistent with previous VNTR LD findings and supports a role for other regional risk variants.

**Table 2:** Individual Haplotype GLM Results.

Haplotype*	Combined Diagnosis vs. Controls				AOS Diagnosis vs. Controls			COS Diagnosis vs. Controls		
	Freq. controls (n = 307)	Freq. patients (n = 691)	P	OR (CI)	Freq. AOS (n = 602)	P	OR (CI)	Freq. COS (n = 89)	P	OR (CI)
TT3T	0.560	0.576	0.035	1.32(1.01–1.69)	0.578	0.034	1.34(1.02–1.75)	0.565	0.327	
TT > 3T	0.251	0.202	Ref.	1	0.202	Ref.	1	0.193	Ref.	1
Rare Group			0.024 RG	2.36(1.12–4.97)		0.013 RG	2.22(1.18–4.18)		0.524 RG	
TT > 3G	0.000	0.002	RG		0.001	RG		0.006	RG	
TG3T	0.000	0.012	RG		0.014	RG		0.000	NA	
TG3G	0.006	0.003	RG		0.003	RG		0.006	RG	
TG > 3T	0.002	0.006	RG		0.007	RG		0.000	RG	
TG > 3G	0.005	0.001	RG		0.000	RG		0.006	RG	
AT3T	0.006	0.017	0.112		0.011	RG		0.052	0.0008	10.28(2.66–39.80)
AT3G	0.002	0.016	0.022	10.49(1.45–81.7)	0.018	0.016	11.81(1.57–88.61)	0.000	RG	
AT > 3T	0.007	0.001	RG		0.002	RG		0.005	RG	
AT > 3G	0.000	0.007	RG		0.007	RG		0.005	RG	
AG3T	0.000	0.001	RG		0.001	RG		0.000	NA	
AG3G	0.108	0.099	0.376		0.094	0.533		0.122	0.321	
AG > 3T	0.000	0.001	RG		0.001	RG		0.000	NA	
AG > 3G	0.053	0.058	0.282		0.061	0.18		0.041	0.717	
Global Haplotype Score			4.9e-03			3.3e-03			2.6e-03	

*SNP order: rs1198588, rs2660304, VNTR, rs1625579).

NA - Haplotypes that were excluded from global analysis due to low frequency (<1%), or that were not found in patients or controls in the haplo.glm analysis.

RG - Haplotypes grouped in the “rare group” glm analysis.

Ref. - the reference haplotype used for the glm analysis.

It is apparent from our data that the VNTR is not the only risk variant within the region as evidenced by low LD with the GWAS SNPs, the lack of risk association with the AG3G haplotype and high risk associations found with the rare AT3G and TG3T haplotypes. The high risk of these rare haplotypes suggest that recombination of the rs2660304 SNP region may also be contributing to risk. In order to evaluate how recombination alone may contribute to risk independent of the VNTR, we conducted global and individual haplotype analyses utilizing only SNP genotypes, excluding the VNTR variant. Results of these analyses confirm that recombination of the rs2660304 SNP alone is a driver of risk (Global haplotype scores: Combined: TGT p = 2.8e-3, ATG p = 1.9e-3; AOS: TGT p = 1.2e-3, ATG p = 9.9e-3. Individual GLM: Combined TGT OR: 12.24 p = 0.015, ATG OR: 12.22 p = 0.015; AOS TGT OR: 14.40 p = 9.0e-3, ATG OR: 13.27 p = 0.012). These results suggest that recombination of the rs2660304 region contributes to risk independently of VNTR genotype, through a presently unknown mechanism.

A different pattern of association was found for COS diagnosis. The rare haplotype AT3T was significant (haplo.glm OR: 10.28, p = 8.0e-4), and no association was found with the common TT3T haplotype. This result is consistent with our previous data demonstrating a genotype association with the minor rs1198588 genotype (A/A) and COS diagnosis. These results may be spurious due to sample differences or they may represent an etiological difference between COS and AOS. Further functional studies will be needed to confirm risk association.

As seen in Table 2, confidence intervals for rare haplotype associations are large due to low haplotype frequencies, larger sample sizes will be needed to determine more accurate effect sizes and replicability of the association findings. Overall, these results demonstrate a complex pattern of risk architecture for the *MIR137HG* region with shorter VNTR lengths and recombination of the rs2660304 region both independently contributing to risk for SZ diagnosis.

### Human specific VNTR expansion

**Figure 5:**
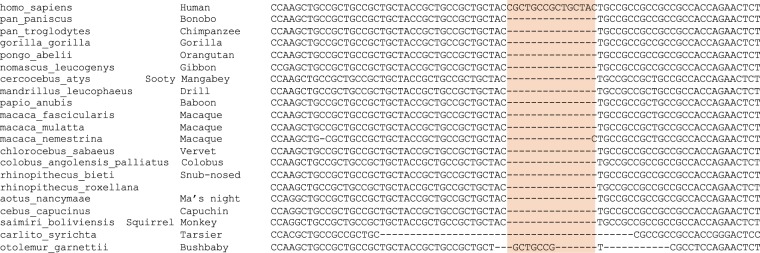
Comparative Genomic Conservation of the VNTR. Sequence alignment of the VNTR across the primate lineage shows high conservation of the region and the expansion from 2 repeats to 3 repeats in the human line. This human lineage specific VNTR expansion may contribute to associations with the *MIR137HG* region and intelligence as well as associations with SZ and ASD.

Previous research demonstrates a significant correlation between VNTR genotype and cognitive phenotypes^22^. We hypothesize that expansion of the VNTR is evolutionarily advantageous for cognition, due to its potential negative regulation of miR-137. To evaluate the VNTR in the context of mammalian evolution we used Ensembl Comparative Genomics to evaluate conservation. Sequence alignment shows that the VNTR length of 2 repeats and the surrounding region is highly conserved throughout the primate lineage (Fig. 5). However, the expansion of the VNTR to 3 repeats is human lineage specific. This result suggests that VNTR expansion may have contributed to increased human cognitive abilities and may contribute to risk for Autism Spectrum Disorder (ASD) as well as SZ^30^.

## Discussion

Through this work, we report novel miR-137 regulatory mechanisms and further characterize SZ risk architecture of the *MIR137HG* genome region. Transcript characterization at early and late developmental stages (fetal vs. adult) revealed a novel pri-miR-137 transcript isoform, del-miR-137. Expression of this lncRNA would down-regulate mature miR-137 expression through alternative 5′ splicing within the pri-miR-137 sequence. Importantly, we found a significant positive association with del-miR-137 transcripts and VNTR length. Increased VNTR lengths were observed more frequently within del-miR-137 transcripts. Analysis of regional GC content demonstrates that the VNTR contributes to length of an overlapping CpG island, which may underlie VNTR associations with alternative splicing. We describe common *MIR137HG* haplotype associations with SZ which are driven by VNTR length, in addition to more rare high risk haplotypes. These associations reveal multiple epistatic risk variants and overall complex risk architecture for the region, that may underlie the common GWAS associations to SZ.

Our present findings are consistent with previous GWAS findings where the common variants are the risk variants. Haplotype analyses revealed risk association with the common TT3T haplotype to increased risk. Critically, our findings demonstrate that the VNTR is a driver of risk only on the background of the common haplotype, as the TT > 3T haplotype imparts a protective effect. In conjunction with our finding that increased VNTR lengths are associated with del-miR-137 splice variants, we predict that the VNTR may mediate risk through altered GC content with the potential to affect DNA binding proteins such as methyl-CpG binding protein 2 (meCP2). MeCP2 has been shown to up-regulate alternative splicing and down-regulate miRNA processing and in this context may contribute to the protective effect of longer VNTR lengths. Functional studies to test this hypothesis are currently underway.

Additionally, we describe a putative novel ceRNA function for the del-miR-137 transcript which may alter axon guidance and other neurodevelopmental pathways and provide additional protective effects for alternatively spliced transcripts independent of reduced miR-137 expression. In general our results suggest that shorter VNTR lengths may contribute to risk for observed miR-137 over-expression in SZ patients^14,17,31–33^ and longer VNTR lengths may impart a protective effect through decreased miR-137 expression. Interestingly, rare high risk haplotypes with recombination of the rs2660304 region were identified. Specific underlying risk mechanisms are unknown, however we predict that cis-acting repressive elements for both the major (TTT) and minor (AGG) haplotypes would be disrupted by recombination. This would result in increased miR-137 expression for the recombined (TGT and ATG) haplotypes, which would contribute to gain-of-function risk for SZ. Replication of our findings in a larger population cohort and further functional analyses is needed to validate these high risk associations and elucidate risk mechanisms.

The human lineage specific expansion of the VNTR supports a potential role for this variant in the evolution of increased cognitive abilities in humans^30^. Longer VNTR repeats would result in a competitive cognitive advantage associated with suppression of miR-137 expression. This is supported by GWAS associations with intelligence and cognitive performance within the *MIR137HG* locus^34–37^. Additionally, VNTR length may contribute to risk for other neurodevelopmental disorders. While SZ and ASD share a subset of phenotypes and risk variants, there are several known pathological variants and pathways in which the direction of risk for SZ is the opposite of risk direction for ASD, also known as the diametric hypothesis^38,39^. The *MIR137HG* locus follows this diametric pattern of association. Findings from GWAS have identified minor allele SNPs within the *MIR137HG* locus to be associated with ASD, which is the opposite finding for GWAS SZ SNPs which are all major alleles^40^. Conditional knockout of miR-137 in mice lead to social deficits and unusual repetitive behavior, phenotypes that are associated with ASD^41^. Individuals with microdeletion of *MIR137HG* had a syndrome presenting with intellectual disability, ASD, and obesity^42–46^. We predict that longer VNTR lengths may confer risk for ASD as miR-137 loss-of-function results in ASD like phenotypes. These findings support an evolutionary role for VNTR expansion in the development of the human brain.

This work contributes to our understanding of the molecular mechanisms underlying the diagnostic associations with common *MIR137HG* genetic variants and SZ. In totality, our findings highlight the complex neurodevelopmental regulation of miR-137 and contribute to our understanding of its role in SZ etiology.

## Methods

### PCR

Complimentary DNA (cDNA) libraries were constructed using standard protocols for the Oligo(dT) primed SuperScript®III First-Strand Synthesis System from commercial human brain tissues (Human Fetal Brain Total RNA from Clontech Cat# 636526 and adult FirstChoice Human Reference Brain Cat# AM6051). Fetal Brain RNA was derived from normal fetal brains pooled from 59 spontaneously aborted male/female Caucasian fetuses, ages: 20–33 weeks. Adult Human Brain RNA was pooled from 21 adult Caucasian individuals. PCR was conducted using custom primer pairs and standard protocols for Platinum™*Taq* DNA Polymerase High Fidelity (ThermoFisher Cat# 11304029). The pri-miR-137 (UCSC ID uc001drx.2) transcript contains 5 exons and 4 introns. Primer pairs were designed with a 5′ guanine (G) to facilitate cloning, and included a forward primer for each of the 5 exons with a reverse primer in exon 5. Primer sequences available on request. Resulting PCR products were run on an agarose gel to verify correct transcript sizes and visualize banding patterns.

### Cloning

Cloning of total PCR products was conducted using the TOPO®TA®Cloning Kit with pCR™2.1-TOPO®vector and One Shot®Chemically competent cells. Platinum *Taq* DNA Polymerase was used for 3′ A-tailing of PCR products to improve cloning efficiency. Cells were plated on either 50 ug/ml kanamycin or 100 ug/ml ampicillin agar plates and blue and white screening was used to select vector containing colonies. Colonies were transfered to a 100 mg/ml ampicillin LB broth starter culture overnight and then 1:500 dilutions were cultured for an additional 13–14 hours. Plasmid DNA was isolated using standard protocols for the QIAprep®Spin Miniprep kit.

### Sequencing

Sequencing of plasmid DNA was conducted through GENEWIZ®using M13 forward and reverse primers. Sequences were aligned to the reference genome using NCBI BLAST®^47^ and CodonCode Aligner software (www.codoncode.com/aligner/index.htm). Novel sequences were deposited to GenBank and assigned the following accession numbers: MK631885, MK631886, MK631887.

### Statistical analyses

#### Sequenced transcript analyses

Associations between spliced transcripts, developmental time points (fetal, adult) and VNTR length were evaluated using Fisher’s exact test (FET), p-values of <0.05 were considered as statistically significant. Transcripts were grouped by age (Adult Brain,Fetal Brain) as well as by VNTR length (3, >3 repeats). Proportions of del-miR-137 transcripts were compared between age groups and between total transcripts grouped by VNTR length. Proportion of del-miR-137 transcripts was compared between VNTR length groups, within age groups (i.e. adult 3R vs. adult >3R). Finally, proportions of del-miR-137 transcripts was compared between age groups keeping VNTR length consistent (i.e. 3R Adult vs. 3R Fetal). Multiple comparisons were corrected for using the Benjamini-Hochberg procedure.

#### Case-control genotype analyses

VNTR length was nominally grouped as either 3R (major allele) or >3R (minor allele) based on genetic and biological findings regarding the VNTR^21,22^ and evaluated using a two-tailed Pearson’s Chi-squared test between combined AOS and COS patients and healthy controls.

For COS genotype associations two-tailed Pearson’s Chi-squared test based on a permutation distribution was used. Fisher’s exact test (FET) with mid-p correction was used to calculate odds ratios (OR) and confidence intervals (CIs). Multiple testing correction was not performed as we considered this a replication analysis of previous associations in SZ^3^; p-values of <0.05 were considered as statistically significant. These results will be used as a point for initial inquiry and not to make determinative conclusions.

### CpG island prediction

EMBOSS Cpgplot was used to evaluate CpG content and putative CpG islands (CGIs) within the *MIR137HG* genome region. Default settings were used: window size = 100, minimum length of an island = 200 bases, minimum average observed/expected ratio of C plus G to CpG = 0.6, minimum average percentage of G plus C = 50.

### Subjects

All study procedures were approved by the Colorado Multiple Institutional Review Board, University of Colorado and samples collected in accordance with the Declaration of Helsinki principles with the appropriate consent. All clinical subjects were drawn from individuals participating in the Department of Psychiatry ‘Genetic Research in Schizophrenia’, part of a larger original study of the Molecular Genetics of Schizophrenia Consortium, and as described previously^48^. A consensus diagnosis of SZ based on DSM-IIIR orDSM-IV criteria was made following a systematic examination of multiple sources of available information obtained from relatives, medical records, clinicians, and direct assessment using one or more diagnostic interviews including the Diagnostic Interview for Genetic Studies (DIGS), the Structured Clinical Interview for Axis I DSM Disorders (SCID). Control subjects were evaluated using the SCID-Non-patient Edition. All subjects provided informed consent to partake in the study. COS subjects were recruited through the Colorado Childhood-onset Schizophrenia Research Program at the University of Colorado School of Medicine^49,50^. Diagnosis of COS was made after the Schedule for Affective Disorders and Schizophrenia for Schoolage Children - Present and Lifetime Version (KSADS- PL) was administered to one parent and the child. Assent by the child, and informed consent was obtained from one or both parents. Criteria for diagnosis were identical to those utilized in adult populations and as described previously^49,50^. All subjects in this study were unrelated to each other. Where postmortem brain DNA samples were used these were obtained from the Schizophrenia Research Center Brain Bank at the University of Colorado, School of Medicine, all of which were donated through the Colorado Anatomical Gift Act and donation approved by the donor family. Ethical approval was obtained for postmortem sample collection and use from the Colorado Multiple Institutional Review Board, University of Colorado and classified as exempt ‘not human tissue research-autopsy’. The Brain Bank contains tissue collected postmortem from subjects with a diagnosis of SZ as well as from unaffected individuals^51^. DNA was isolated using standard methods, from unrelated Caucasian individuals (AOS (n = 602) COS subjects (n = 89) and control (n = 307); See Table 3 for demographics). Subjects were included for analyses only if all variants were successfully genotyped.

**Table 3:** Subject Demographics.

Subjects	N	Mean Age* +/− SE	No. (%) male
Controls	307	51.0 +/− 0.85	161 (52.4%)
COS	89	10.1 +/− 0.28	61 (68.5%)
AOS	602	42.2 +/− 0.53	421 (69.9%)

COS- childhood onset schizophrenia; AOS- adult onset schizophrenia.

*Age at collection is given for samples from blood draws, age at death is given for brain samples, age at first visit to clinic typically coinciding with age of diagnosis is given for COS samples.

### Genotyping

Three GWAS significant SNPs (rs1625579, rs1198588, rs2660304) were genotyped for all individuals. All SNPs were in Hardy-Weinberg equilibrium in controls (rs1625579: *χ*^2^ = 0.275, p = 0.600; rs1198588: *χ*^2^ = 0.039, p = 0.844; rs2660304: *χ*^2^ = 0.275, p = 0.600). Genotyping was performed in duplicate for each sample using TaqMan Genotyping Assays and qPCR on a 7900HT Fast Real-Time PCR System. Samples were excluded if genotype calls were undetermined or if discrepant calls were made between duplicates after two rounds of genotyping. Multiple testing correction was not performed as we considered this a replicative analysis of previous associations in SZ^1,2,52^; p-values of <0.05 were considered as statistically significant.

An optimized PCR protocol for the VNTR was used to genotype all individuals with custom VNTR primers (Forward: 5′ GCTCAGCGAGCAGCAAGAGT; Reverse: 5′ GTCACCGAAGAGAGTCAGAGGACC) using the GC-RICH PCR System mfr. Roche. PCR products were run on a 3% agarose gel to resolve the 15 bp repeat size differences. After a sufficient number of samples were run, a band size ladder was generated with VNTR lengths ranging from 3–12 repeats. Ladder results were confirmed through DNA Fragment analysis (GENEWIZ) with a fluorescent FAM labeled primer (FAM labeled: 5′ TCACCGAAGAGAGTCAGAGGACC). Manual calls were 100% consistent with fragment analysis results. This ladder was used for comparison to genotype remaining individuals manually. Linkage disequilibrium (LD) between the VNTR and GWAS SNPs was calculated in the control cohort using the R package ‘genetics’ LD function.

### Haplotype analyses methods

Haplotype analysis was conducted using R haplo.score and haplo.glm functions^53^. Input data included unphased genotypes for all GWAS SNPs (rs1625579, rs1198588, rs2660304) and the VNTR for each individual. Global score tests of association use an additive GLM to regress diagnosis on haplotype pairs for an individual. Correction for multiple testing was performed by applying the simulate = TRUE parameter in haplo.score which provides simulated *p* values for each haplotype. Haplotypes were excluded with frequencies <0.8% for the combined diagnosis group, and <1% for AOS and COS groups. The magnitude and direction of individual haplotype effects is determined using a Wald type test relative to a common reference haplotype. Initially, rare haplotypes (<0.8%) were grouped and included in the model. As complete separation was seen for the TG3T haplotype, minimum haplotype frequency was raised to 0.9% to include the TG3T haplotype with in the rare group and better fit the model. In order to evaluate effects the common haplotype, the second most frequent haplotype was used as the reference. An additional haplotype analysis was conducted using only GWAS SNPs to evaluate VNTR independent associations.

### VNTR conservation

Ensembl (release 95) Comparative Genomics was used to evaluate the conservation of the VNTR in the 75 eutherian mammals alignment set. The evaluated sequence included GRCh38.p12 Chromosome 1: 98,046,166-98,046,254.

### ceRNA pathway

The miRDB.org website was used to generate a list of predicted miRs that bind to the del-miR-137 transcripts^25^. The miRDB tool uses a custom target prediction algorithm, MirTarget v4.0, which combines CLIP binding data and miRNA overexpression data to predict microRNA binding sites^54^. The algorithm assigns target prediction scores between 50–100. Per miRDB recommendations, only miRNAs with a target score >60 were used for further analysis. This list of miRNAs was then input into the miRSystem to determine potential target genes and conduct pathway ranking analysis for target gene pathways^26^. MiRSystem utilizes 7 algorithms predicting miRNA targets and two experimentally validated databases of miRNA target genes. Pathway analysis results are based solely on experimentally validated miR/target gene pairs.

## Supplementary information

supplementary information

## Data Availability

The cDNA sequences generated and analyzed during the current study are available in the Genbank repository, accession codes: MK631885, MK631886 and MK631887. Other datasets generated and analyzed during the current study are available from the corresponding author on request.
