# Supplementary material for: A VNTR Regulates miR-137 Expression Through Novel Alternative Splicing and Contributes to Risk for Schizophrenia
Source: Sci Rep. 2019 Aug 13;9:11793. doi: 10.1038/s41598-019-48141-0 (PMC6692358; doi:10.1038/s41598-019-48141-0)
Supplement: Supplementary file 1 — supplementary information [file 41598_2019_48141_MOESM1_ESM.pdf]

A VNTR Regulates miR-137 Expression Through Novel Alternative Splicing and Contributes to Risk for Schizophrenia

Ashley Pacheco[1], Ralph Berger[1], Robert Freedman[1], Amanda J. Law[1,2,3*]

From the Departments of Psychiatry[1], Medicine[2] and Cell and Developmental Biology[3], University of Colorado, School of Medicine, Aurora, Colorado 80045.

[*]To whom correspondence should be addressed: Prof. Amanda J. Law, Department of Psychiatry, University of Colorado, School of Medicine, 12700 East 19th Avenue, Mailstop 8619, RC2, RM 4100C, Aurora, Colorado 80045, Telephone: (303) 724-4418; Fax: (303) 724-4425; amanda.law@ucdenver.edu

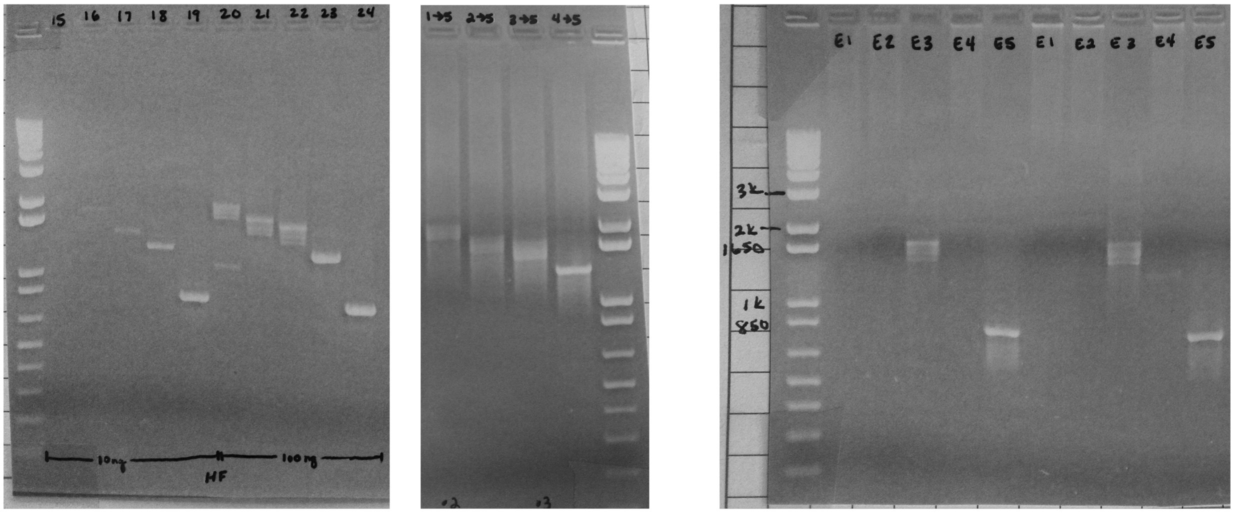

Supplementary Figure S1. Full length PCR gels for custom primer pairs are shown to supplement manuscript Figure 1. The lefthand gel is the result of miR-137 custom primers in adult total brain cDNA as seen in Figure 1a. The center gel is the result of miR-137 custom primers in fetal total brain cDNA as seen in Figure 1a. In the righthand gel lane 3 is the primer pair exon 3 to 5 in adult total brain cDNA and lane 8 is the same primer pair in fetal total brain cDNA as seen in Figure 1b.

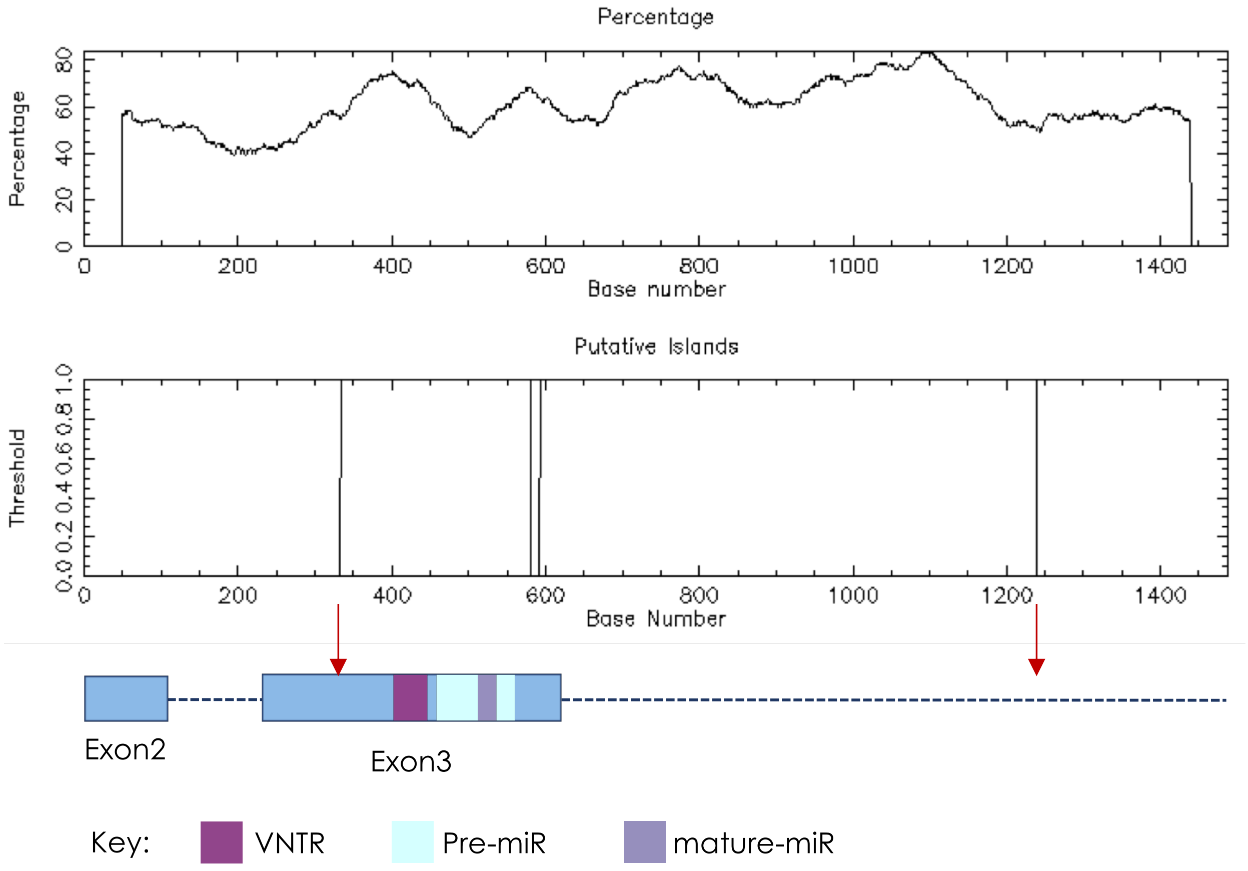

Supplementary Figure S2. EMBOSS CpG island prediction output. The top box displays the percentage of C or G nucleotides across the genome region. The bottom box displays the bounds of predicted CpG islands. Red arrows point to corresponding *MIR137HG* genome location. The CpG islands span the majority of pri-miR-137 exon 3 including the VNTR, pre- and mature miR sequences and continues into intron 3. Increased VNTR repeats would extend the length of the associated CpG island.

**Target Rank**	**Target Score**	**miRNA Name**
1	85	hsa-miR-3123
2	82	hsa-miR-3145-5p
3	81	hsa-miR-4778-3p
4	79	hsa-miR-6806-5p
5	75	hsa-miR-526a*
6	75	hsa-miR-523-5p
7	75	hsa-miR-522-5p
8	75	hsa-miR-520c-5p*
9	75	hsa-miR-519c-5p*
10	75	hsa-miR-519b-5p*
11	75	hsa-miR-519a-5p
12	75	hsa-miR-518f-5p
13	75	hsa-miR-518e-5p
14	75	hsa-miR-518d-5p*
15	75	hsa-miR-4277
16	72	hsa-miR-550a-5p*
17	72	hsa-miR-550a-3-5p
18	72	hsa-miR-7109-5p
19	71	hsa-miR-634*
20	70	hsa-miR-4257
21	70	hsa-miR-6742-5p
22	69	hsa-miR-650*
23	69	hsa-miR-3612
24	66	hsa-miR-378g
25	66	hsa-miR-944*
26	64	hsa-miR-802*
27	64	hsa-miR-4427
28	63	hsa-miR-1285-5p
29	62	hsa-miR-7852-3p
30	62	hsa-miR-3180-5p
31	61	hsa-miR-4762-5p
32	59	hsa-miR-651-3p
33	58	hsa-miR-4446-5p
34	58	hsa-miR-4443
35	57	hsa-miR-4251
36	57	hsa-miR-4639-5p
37	56	hsa-miR-4263
38	56	hsa-miR-519e-3p
39	56	hsa-miR-515-3p
40	56	hsa-miR-33b-3p
41	55	hsa-miR-491-5p
42	55	hsa-miR-6777-3p
43	55	hsa-miR-4457
44	54	hsa-miR-509-5p
45	54	hsa-miR-509-3-5p
46	54	hsa-miR-4418
47	53	hsa-miR-455-3p
48	53	hsa-miR-4758-3p
49	52	hsa-miR-3168
50	52	hsa-miR-7515
51	52	hsa-miR-186-5p
52	51	hsa-miR-3667-3p
53	50	hsa-miR-519d-5p
54	50	hsa-miR-8063
55	50	hsa-miR-4743-3p
56	50	hsa-miR-1296-3p

*validated miRs in the axon guidance pathway

Supplementary Table S1. Del-miR-137 Predicted Binding miRNAs predicted using miRDB.org, Wong and Wang (2015)

Target Gene	Gene Description
ANK2	ankyrin 2 neuronal
ARHGEF12	Rho guanine nucleotide exchange factor (GEF) 12
CACNB4	calcium channel voltage-dependent beta 4 subunit
CLASP2	cytoplasmic linker associated protein 2
CNTNAP1	contactin associated protein 1
COL1A1	collagen type I alpha 1
COL4A4	collagen type IV alpha 4
COL5A1	collagen type V alpha 1
COL5A2	collagen type V alpha 2
CREB1	cAMP responsive element binding protein 1
CRMP1	collapsin response mediator protein 1
DCX	doublecortin
DPYSL2	dihydropyrimidinase-like 2
DPYSL5	dihydropyrimidinase-like 5
ENAH	enabled homolog (Drosophila)
FGFR1	fibroblast growth factor receptor 1
ITGAV	integrin alpha V
KIAA1598	Shootin-1
MYO10	myosin X
NRAS	neuroblastoma RAS viral (v-ras) oncogene homolog
NRCAM	neuronal cell adhesion molecule
NRP1	neuropilin 1
NTN4	netrin 4
PAK2	p21 protein (Cdc42/Rac)-activated kinase 2
PAK7	p21 protein (Cdc42/Rac)-activated kinase 7
PFN2	profilin 2
RAC1	ras-related C3 botulinum toxin substrate 1 (rho family small GTP binding protein Rac1)
ROBO1	roundabout axon guidance receptor homolog 1 (Drosophila)
ROBO2	roundabout axon guidance receptor homolog 2 (Drosophila)
RPS6KA1	ribosomal protein S6 kinase, 90kDa, polypeptide 1
RPS6KA3	ribosomal protein S6 kinase, 90kDa, polypeptide 3
SCN1A	sodium channel, voltage-gated, type I, alpha subunit
SCN3A	sodium channel, voltage-gated, type III, alpha subunit
SEMA3A	sema domain, immunoglobulin domain (Ig), short basic domain, secreted, (semaphorin) 3A
SEMA6A	sema domain, transmembrane domain (TM), and cytoplasmic domain, (semaphorin) 6A
SEMA6D	sema domain, transmembrane domain (TM), and cytoplasmic domain, semaphorin) 6D
SIAH1	siah E3 ubiquitin protein ligase 1
SPTBN1	spectrin, beta, non-erythrocytic 1
SRGAP3	SLIT-ROBO Rho GTPase activating protein 3
ST8SIA2	ST8 alpha-N-acetyl-neuraminide alpha-2, 8-sialyltransferase 2
ST8SIA4	ST8 alpha-N-acetyl-neuraminide alpha-2, 8-sialyltransferase 4
TRPC3	transient receptor potential cation channel, subfamily C, member 3
TRPC5	transient receptor potential cation channel, subfamily C, member 5
UNC5D	unc-5 homolog D (C. elegans)
WASL	Wiskott-Aldrich syndrome-like

Supplementary Table S2. Target genes for the Del-miR binding miRs found in the 'Axon Guidance' pathway. Predicted by miRSystem (Tzu-Pin Lu et. al., 2012).

	Controls(n=306)		Combined AOS/COS (n=678)		AOS(n=590)		COS(n=88)	
**Allele**	**n**	**Freq.(%)**	**n**	**Freq.(%)**	**n**	**Freq.(%)**	**n**	**Freq.(%)**
3	417	68.1	980	72.3	849	71.9	131	74.4
4	71	11.6	121	8.9	103	8.7	18	10.2
5	30	4.9	65	4.8	61	5.2	4	2.3
6	16	2.6	44	3.2	42	3.6	2	1.1
7	21	3.4	39	2.9	34	2.9	5	2.8
8	19	3.1	30	2.2	23	1.9	7	4.0
9	13	2.1	30	2.2	24	2.0	6	3.4
10	11	1.8	28	2.1	25	2.1	3	1.7
11	10	1.6	10	0.7	10	0.8	-	-
12	1	0.2	7	0.5	7	0.6	-	-
13	3	0.5	-	-	-	-	-	-
14	-	-	2	0.1	2	0.2	-	-
Total	612		1356		1180		176	
**Genotype**	**n**	**Freq.**	**n**	**Freq.**	**n**	**Freq.**	**n**	**Freq.**
3/3	143	46.7	346	51.0	297	50.3	49	55.7
3/>3	131	42.8	288	42.5	255	43.2	33	37.5
>3/>3	32	10.5	44	6.5	38	6.4	6	6.8
**Genotype**	**n**	**Freq.**	**n**	**Freq.**	**n**	**Freq.**	**n**	**Freq.**
3/3	143	46.7	346	51.0	297	50.3	49	55.7
3/4	50	16.3	91	13.4	78	13.2	13	14.8
3/5	19	6.2	45	6.6	44	7.5	1	1.1
3/6	8	2.6	31	4.6	30	5.1	1	1.1
3/7	19	6.2	30	4.4	26	4.4	4	4.5
3/8	11	3.6	24	3.5	18	3.1	6	6.8
3/9	8	2.6	25	3.7	20	3.4	5	5.7
3/10	10	3.3	24	3.5	21	3.6	3	3.4
3/11	4	1.3	9	1.3	9	1.5	-	-
3/12	1	0.3	7	1.0	7	1.2	-	-
3/13	1	0.3	-	-	-	-	-	-
3/14	-	-	2	0.3	2	0.3	-	-
4/4	1	0.3	6	0.9	4	0.7	2	2.3
4/5	4	1.3	4	0.6	3	0.5	1	1.1
4/6	3	1.0	4	0.6	4	0.7	-	-
4/7	-	-	4	0.6	4	0.7	-	-
4/8	3	1.0	3	0.4	3	0.5	-	-
4/9	4	1.3	1	0.1	1	0.2	-	-
4/10	-	-	2	0.3	2	0.3	-	-
4/11	4	1.3	-	-	-	-	-	-
4/13	1	0.3	-	-	-	-	-	-
5/5	2	0.7	2	0.3	2	0.3	-	-
5/6	1	0.3	4	0.6	4	0.7	-	-
5/7	-	-	3	0.4	3	0.5	-	-
5/8	1	0.3	3	0.4	2	0.3	1	1.1
5/9	-	-	1	0.1	-	-	1	1.1
5/10	-	-	1	0.1	1	0.2	-	-
5/13	1	0.3	-	-	-	-	-	-
6/6	1	0.3	1	0.1	1	0.2	-	-
6/7	1	0.3	1	0.1	-	-	1	1.1
6/9	1	0.3	-	-	-	-	-	-
6/10	-	-	1	0.1	1	0.2	-	-
6/11	-	-	1	0.1	1	0.2	-	-
7/9	-	-	1	0.1	1	0.2	-	-
7/11	1	0.3	-	-	-	-	-	-
8/8	1	0.3	-	-	-	-	-	-
8/10	1	0.3	-	-	-	-	-	-
8/11	1	0.3	-	-	-	-	-	-
9/9	-	-	1	0.1	1	0.2	-	-

- Dashes indicate variants not found in the diagnostic group

Supplementary Table S3. VNTR allele and genotype results for all subjects.
